# Supplementary material for: Giant viruses specific to deep oceans show persistent presence and activity
Source: mSystems. 2025 Nov 12;10(12):e00932-25. doi: 10.1128/msystems.00932-25 (PMC12710325; doi:10.1128/msystems.00932-25)
Supplement: Supplemental Figures — Fig. S1 to S16. [file msystems.00932-25-s0001.docx]

**Giant viruses specific to deep oceans show persistent presence and activity**

Wenwen Liu, Komei Nagasaka, Junyi Wu, Hiroki Ban, Ethan Mimick, Lingjie Meng, Russell Y. Neches, Mohammad Moniruzzaman, Takashi Yoshida, Yosuke Nishimura, Hisashi Endo, Yusuke Okazaki, Hiroyuki Ogata

**Supplementary Figures**


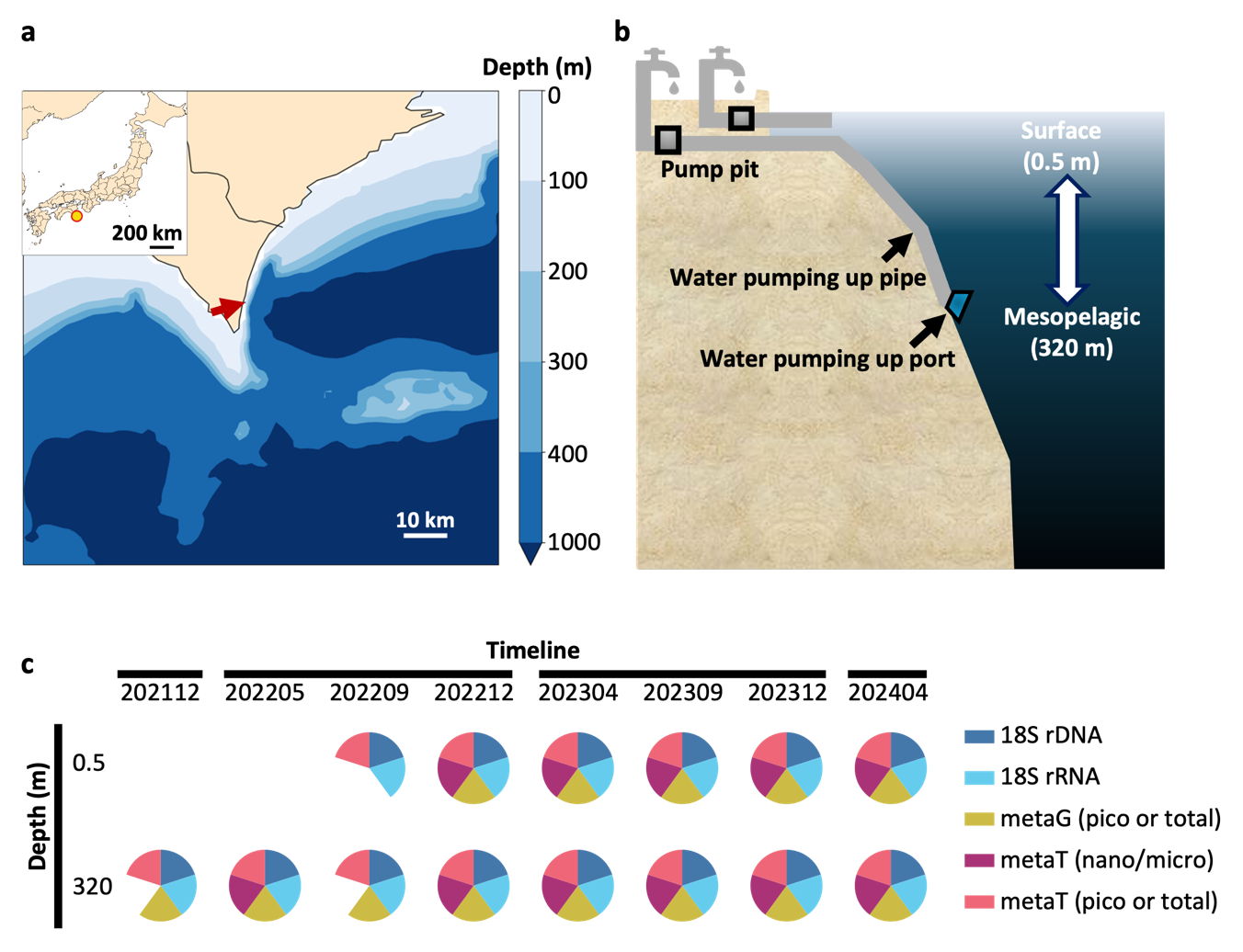


Figure S1. Sampling location and depth. (a) Location of the sampling site at the Kochi Prefectural Deep Seawater Laboratory. (b) Schematic illustration of surface and mesopelagic seawater intake equipment. (c) Diagram showing the sampling year and month as well as depth, either surface (0.5 m) or mesopelagic (320 m). 18S rDNA: DNA-based 18S rRNA metabarcoding; 18S rRNA: cDNA-based 18S rRNA metabarcoding; metaG (pico or total): metagenomic sequencing of the pico- or total-fraction; metaT (nano/micro): metatranscriptomic sequencing of the nano/micro-fraction; metaT (pico or total): metatranscriptomic sequencing of the pico or total fraction.


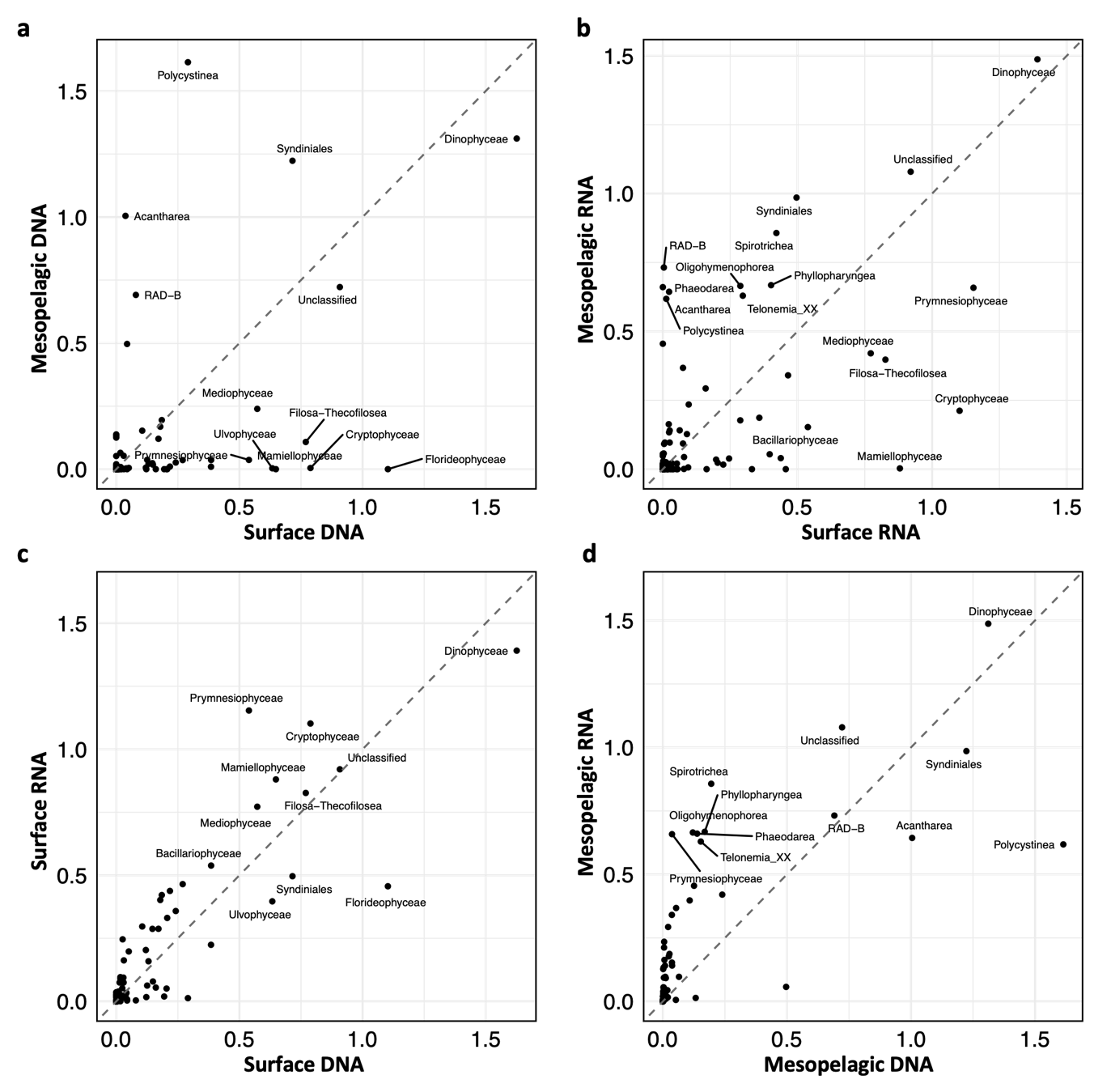


Figure S2. Comparisons of mean relative abundance (logTPM+1) of taxa at class level classification based on 18S rDNA/rRNA metabarcoding data. The taxa with logTPM+1 > 0.5 are labeled.


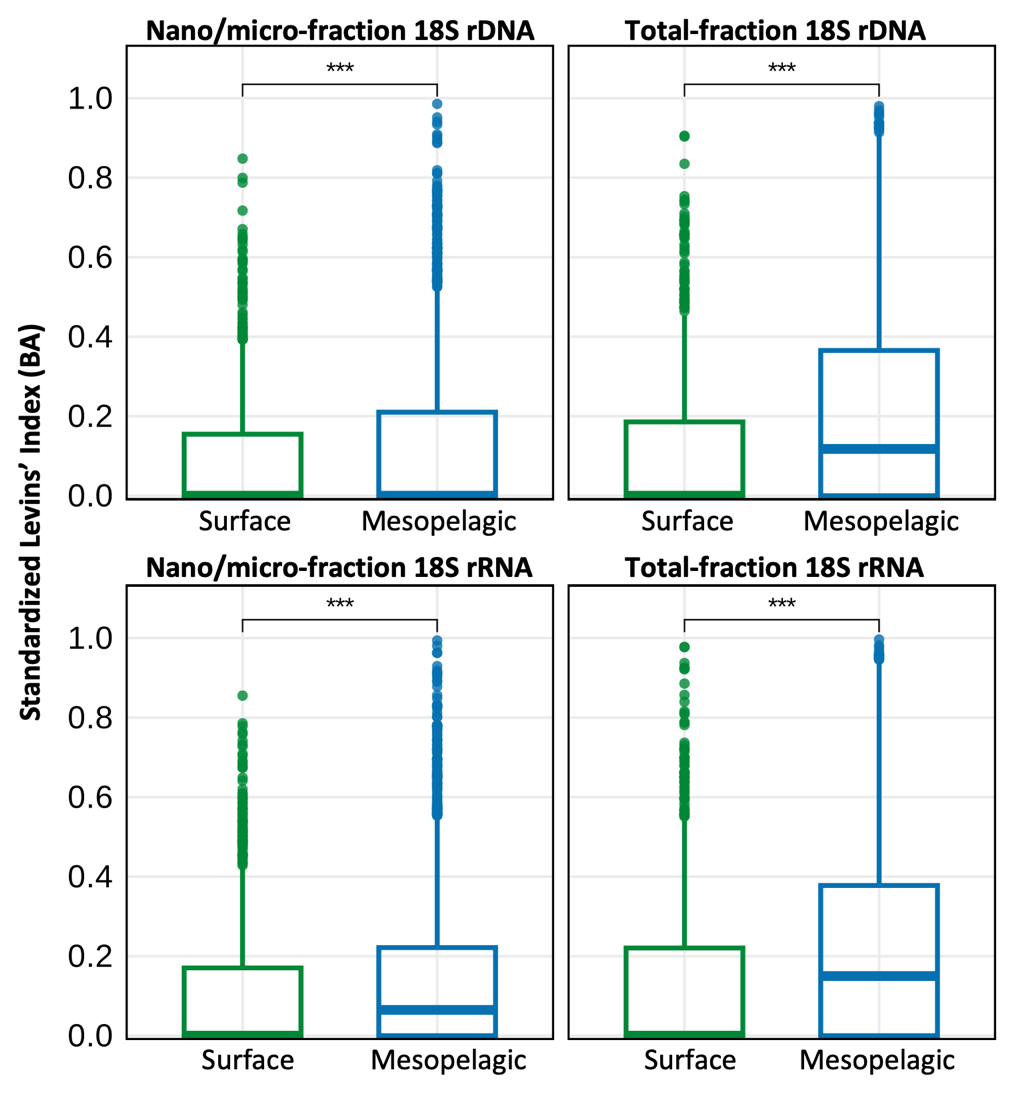


Figure S3. Comparison of Levins’ standardized niche breadth (****BA****) between ****surface**** and ****mesopelagic**** ASVs****.**** Boxes show medians and interquartile ranges; whiskers extend to 1.5×IQR; points denote outliers. The Wilcoxon rank-sum test was used for statistical tests. Asterisks (***) indicate *P* ***<* 0.001**.


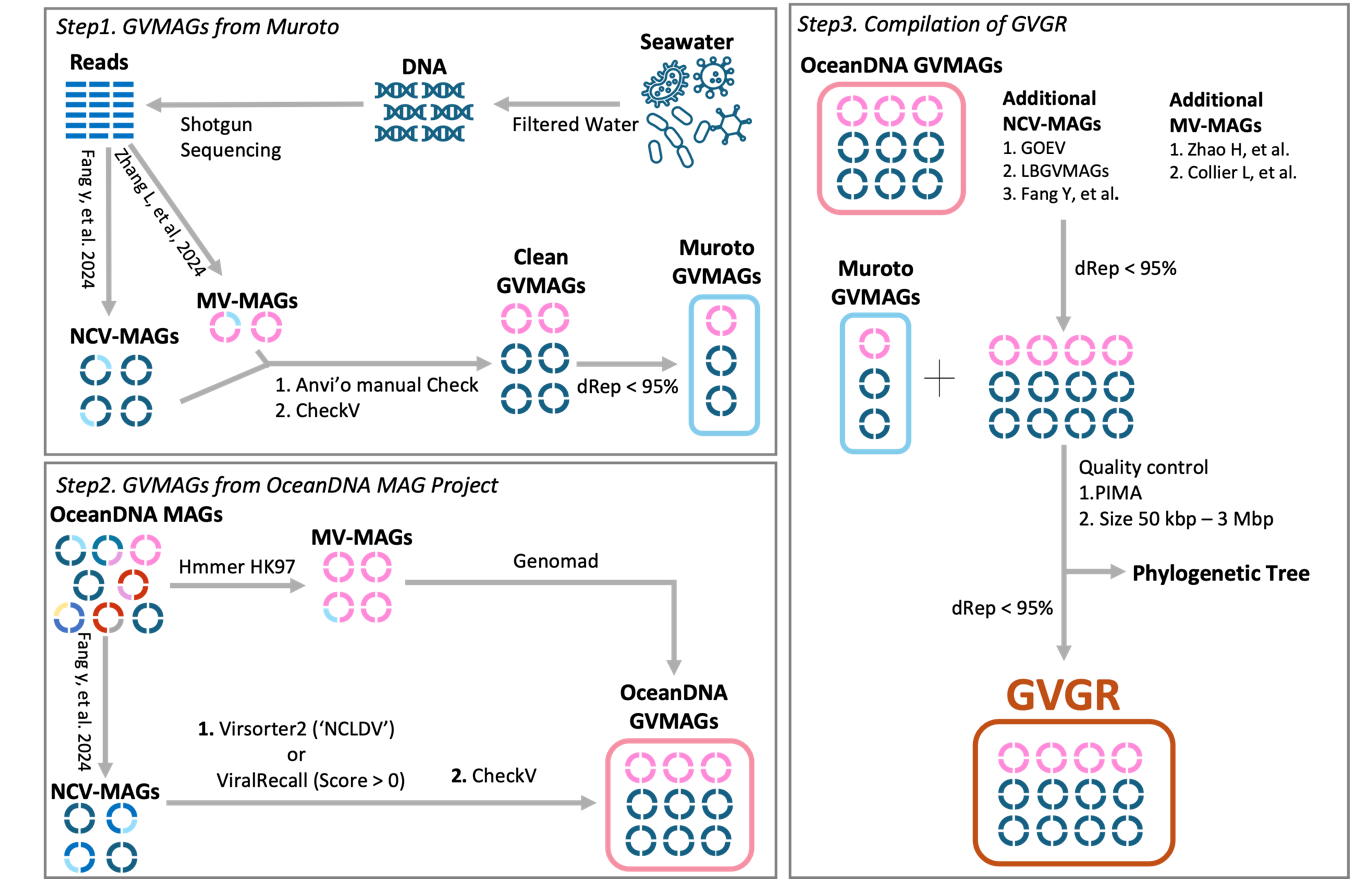


Figure S4. Pipeline for generating the giant virus genome reference (GVGR) database. The pipeline consists of three steps: (1) manually curated GVMAGs (Muroto GVMAGs) generated from Muroto metagenomic data in this study; (2) GVMAGs (OceanDNA GVMAGs) extracted from the OceanDNA MAG project; and (3) integration of Muroto GVMAGs, OceanDNA GVMAGs, and previously published GVMAG datasets to establish the comprehensive GVGR database.


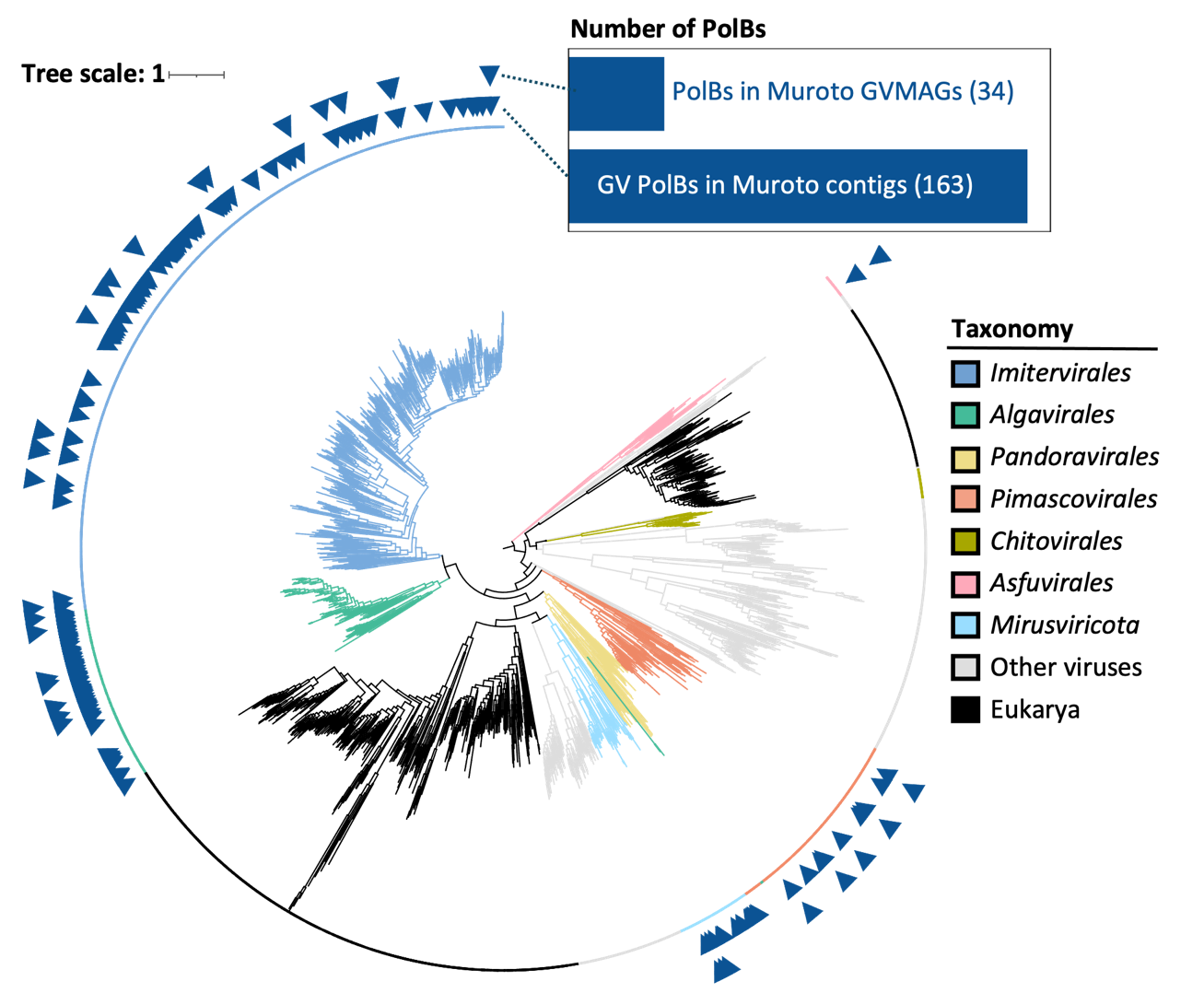


**Figure S5. Phylogenetic analysis of PolB from the Muroto samples.** Maximum-likelihood phylogenetic tree of PolB from Muroto metagenomic contigs (>2.5 kbp) and reference sequences (Gaïa et al., 2023; Kazlauskas et al., 2020). Branch colors and the innermost ring indicate viral classification. The two surrounding rings with triangles indicate the distribution of PolB sequences: the inner ring shows PolB sequences likely to be of GV origin and found in the Muroto contigs (>2.5 kbp), and the outer ring represents PolB sequences found in qualified Muroto GVMAGs. The accompanying bar plot shows the number of PolB sequences corresponding to each ring.

**
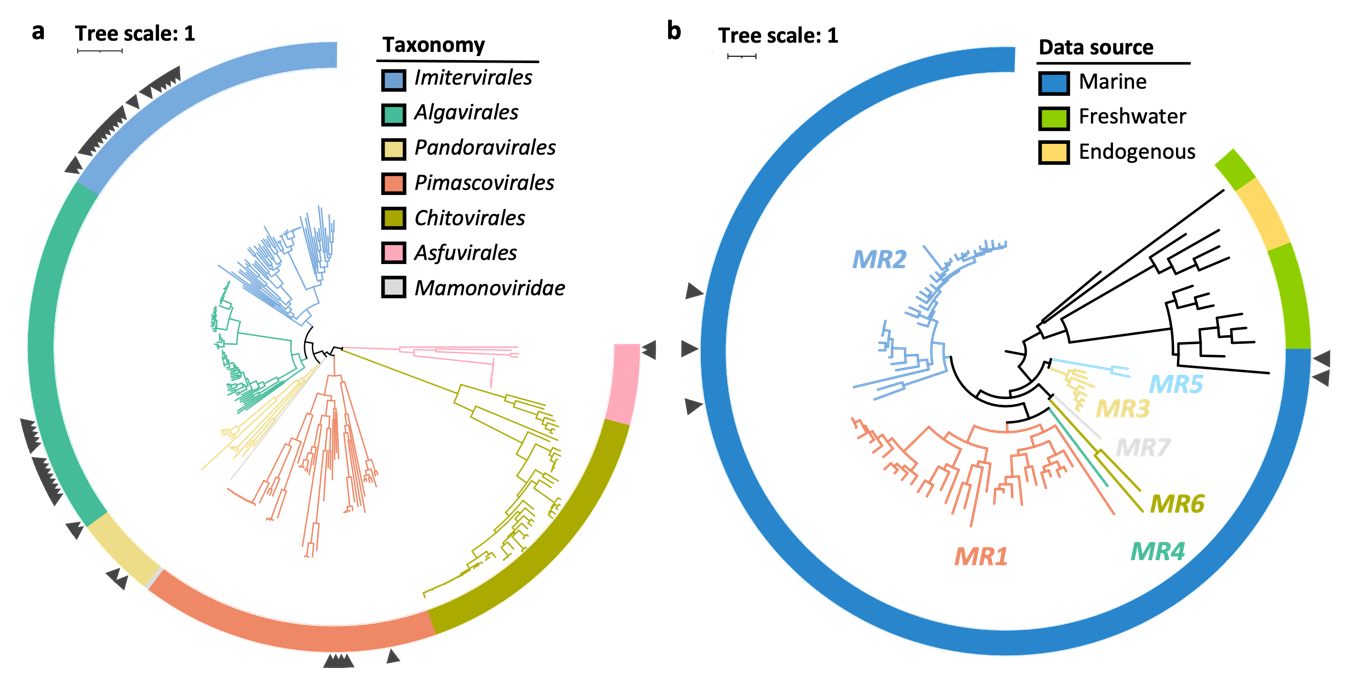
**

**Figure S6. Phylogenetic and taxonomic composition of Muroto GVMAGs.** (a) Maximum-likelihood phylogenetic tree of Muroto NCV-MAGs and 220 reference genomes inferred with IQ-TREE using seven conserved marker genes. Ring and branch colors indicate taxonomic classifications. (b) Phylogenetic tree of Muroto MV-MAGs and reference genomes inferred with IQ-TREE using the HK97 MCP gene. The ring indicates the MAG source, and branch colors represent clade classifications. MR1 to MR6 mark previously described marine mirusvirus clades. Gray triangles indicate Muroto GVMAGs.


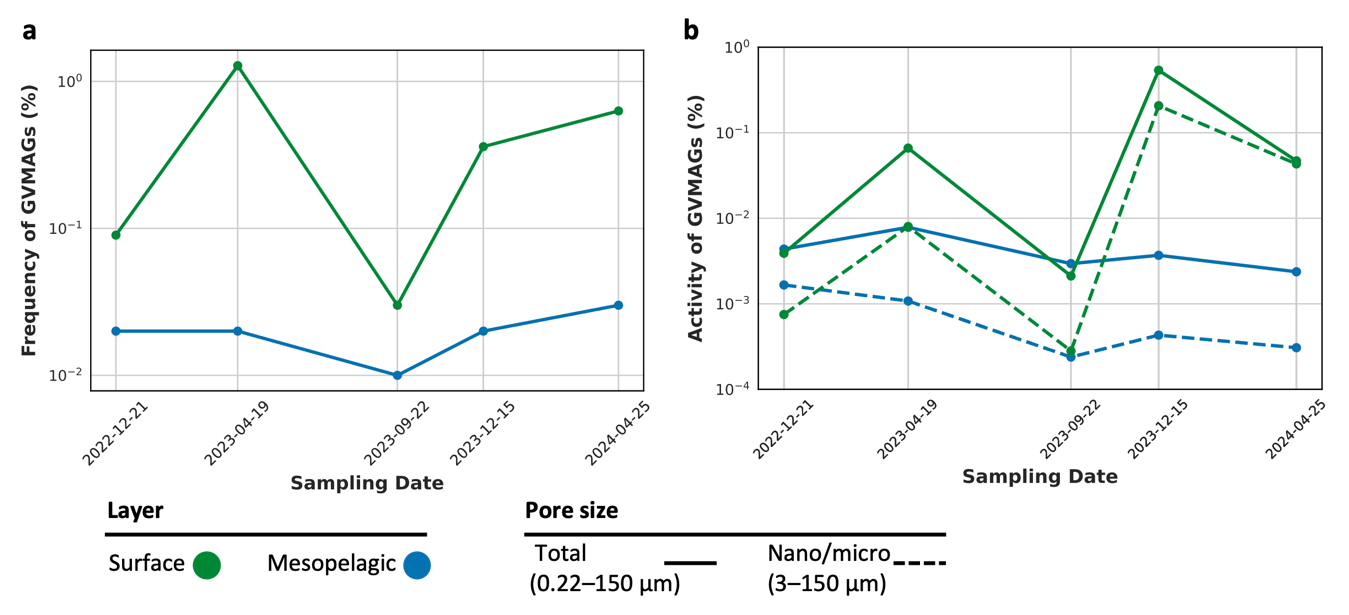


Figure S7. Dynamics of the presence and activity of Muroto GVMAGs. (a) Percentage of metagenomic reads mapped to the 48 Muroto GVMAGs over time. (b) Percentage of metatranscriptomic reads mapped to the 48 Muroto GVMAGs over time. Blue lines represent mesopelagic samples, while green lines represent surface samples. Solid lines indicate total-fractions (0.2–150 µm), whereas dashed lines correspond to nano/micro-fractions (5–150 µm). Time points are plotted based on actual sampling intervals, thus the spacing on the X-axis reflecting real temporal distances.


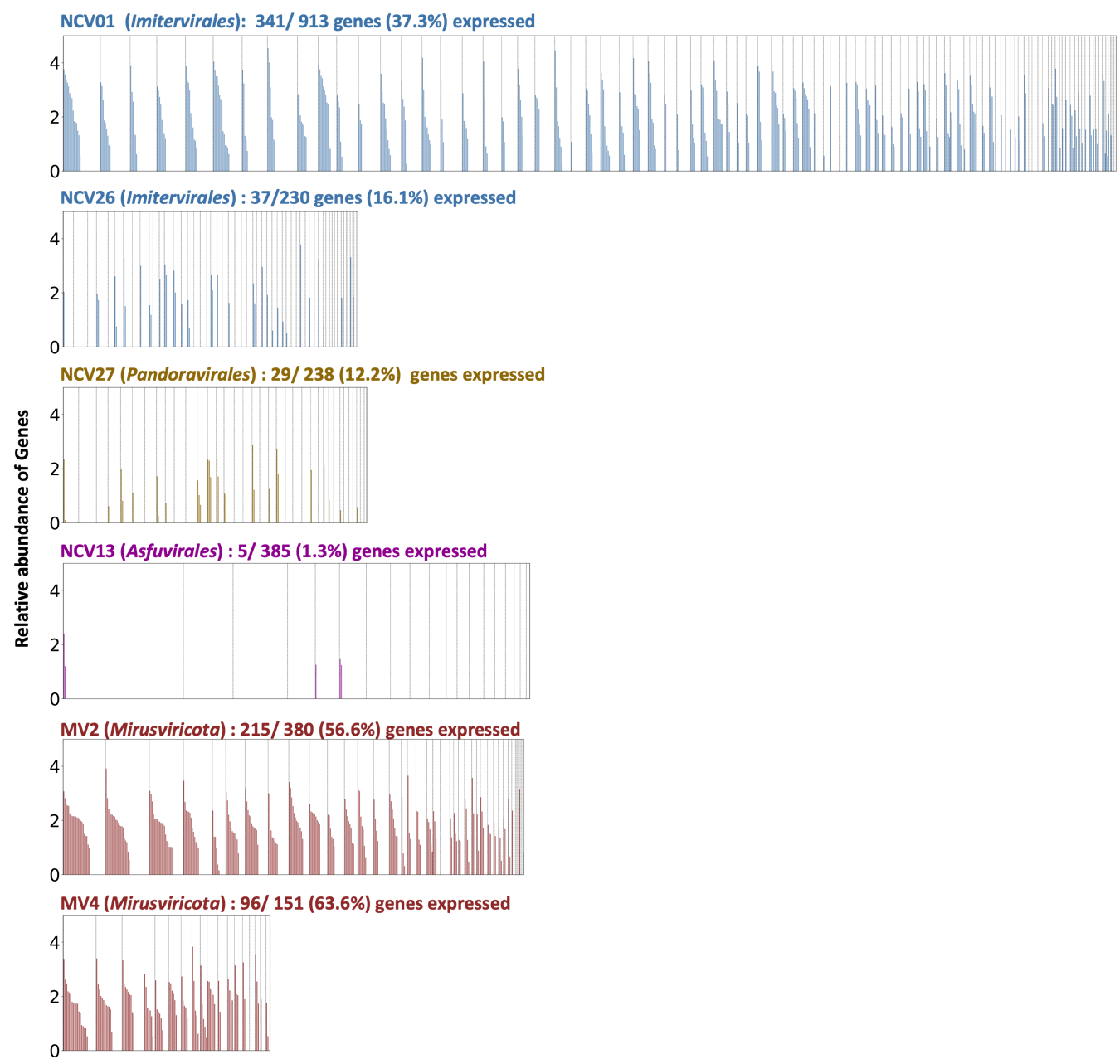


Figure S8. Gene expression profiles of six meso-exclusive GVMAGs. Each horizontal panel represents one meso-exclusive GVMAG, with the X-axis showing individual genes and the Y-axis indicating mean relative levels of transcription (logTPM+1). Bar colors distinguish different GV orders. Vertical dashed lines indicate the boundary between contigs. Within individual contigs, genes are ordered by their expression levels. The total number of genes and the percentage of expressed genes are annotated for each GVMAG.


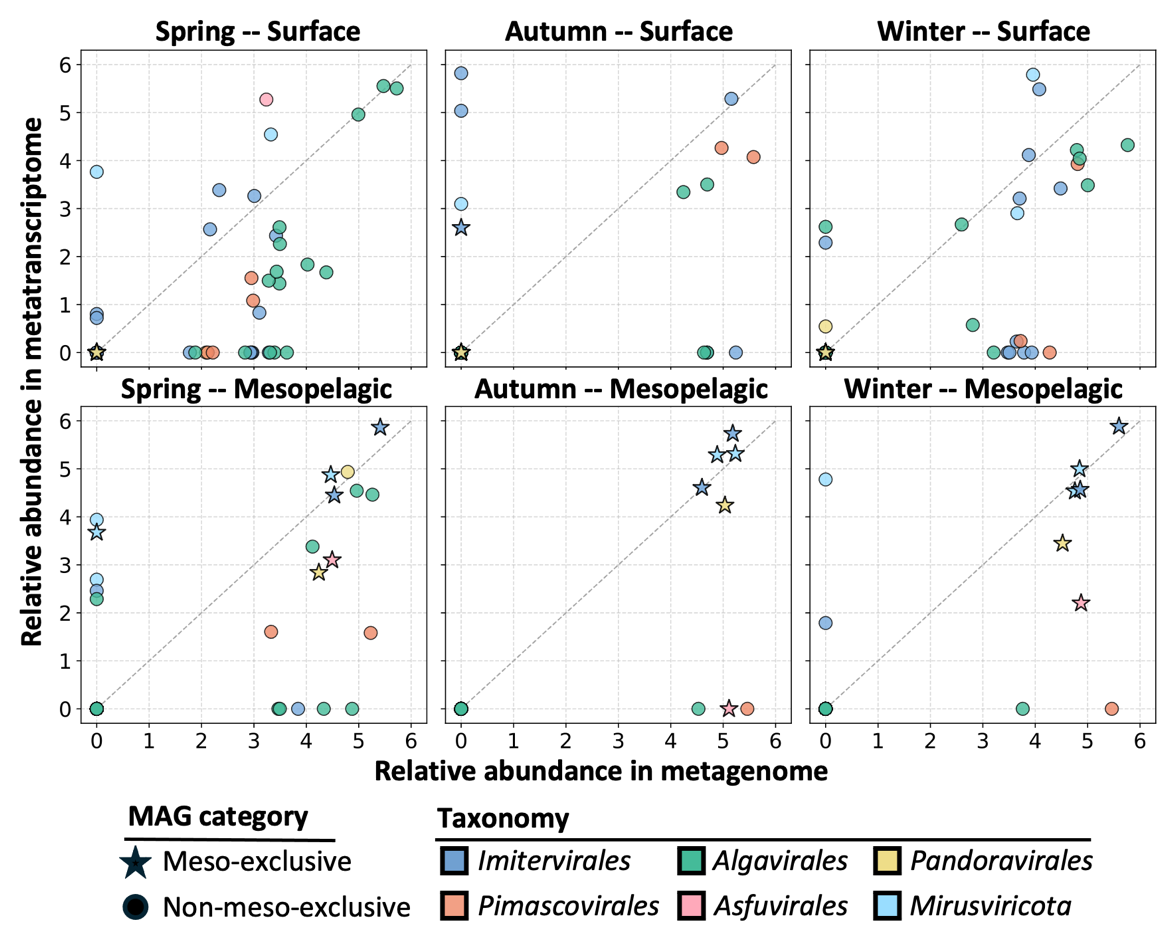


Figure S9. The relative abundance of Muroto GVMAGs between metagenomic and metatranscriptomic datasets across seasons and depths. Scatterplots show the relationship between average relative abundance in metagenomic (X-axis, logTPM+1) and metatranscriptomic (Y-axis, logTPM+1) datasets for Muroto GVMAGs. Columns represent samples from different seasons (spring, autumn, winter), and rows correspond to samples from different depths (surface and mesopelagic). Point color indicates taxonomic classification, and shape denotes MAG categories: stars represent meso-exclusive GVMAGs, while circles indicate non-meso-exclusive GVMAGs.


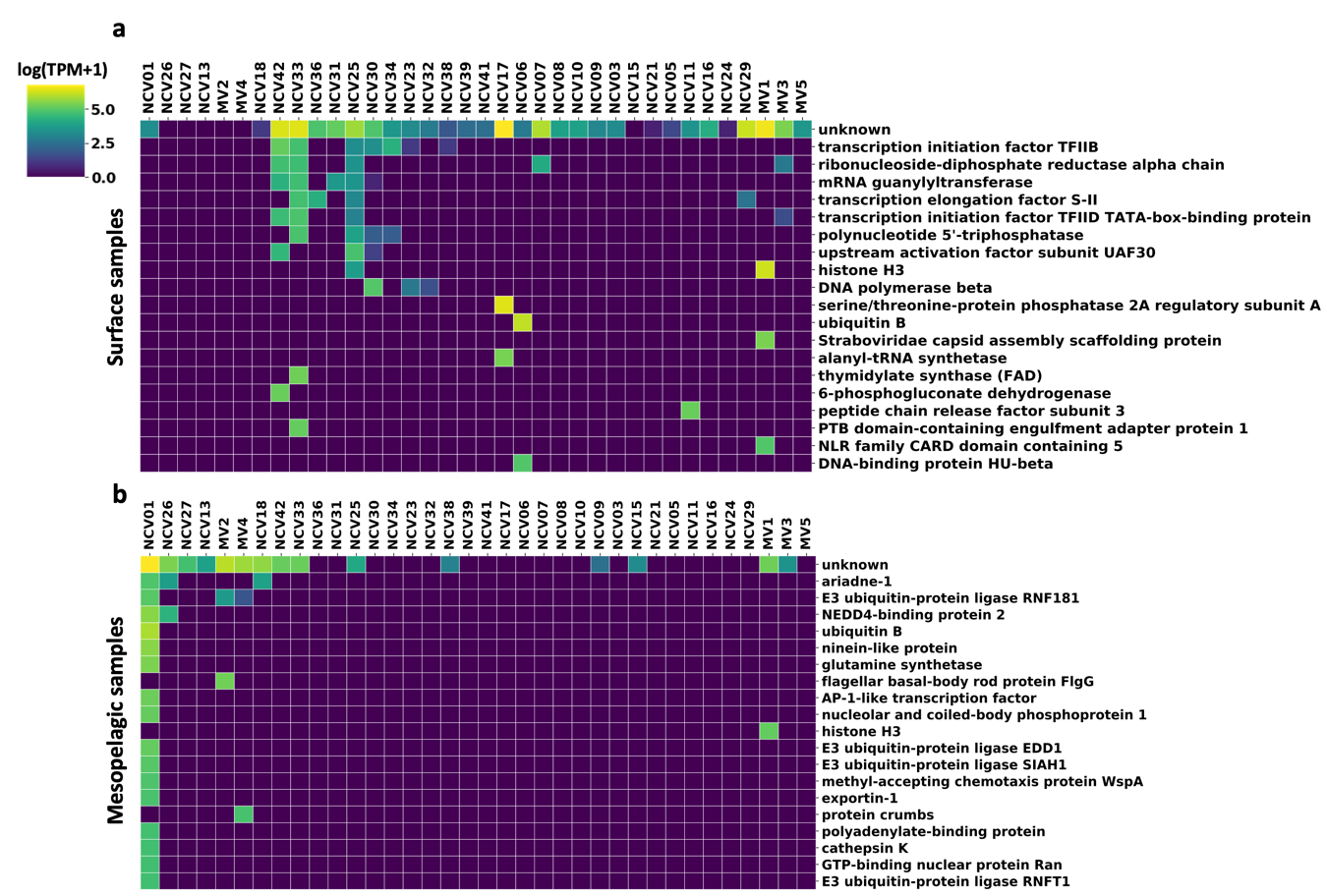


Figure S10. Top 20 higly expressed KEGG orthology (KO) terms for the Muroto GVMAGs. Heatmaps display the mean relative abundance (logTPM+1) of the top 20 most highly expressed KO terms of 48 Muroto GVMAGs in (a) surface and (b) mesopelagic metatranscriptomic datasets. Columns represent GVMAGs, while rows correspond to KO terms.

**
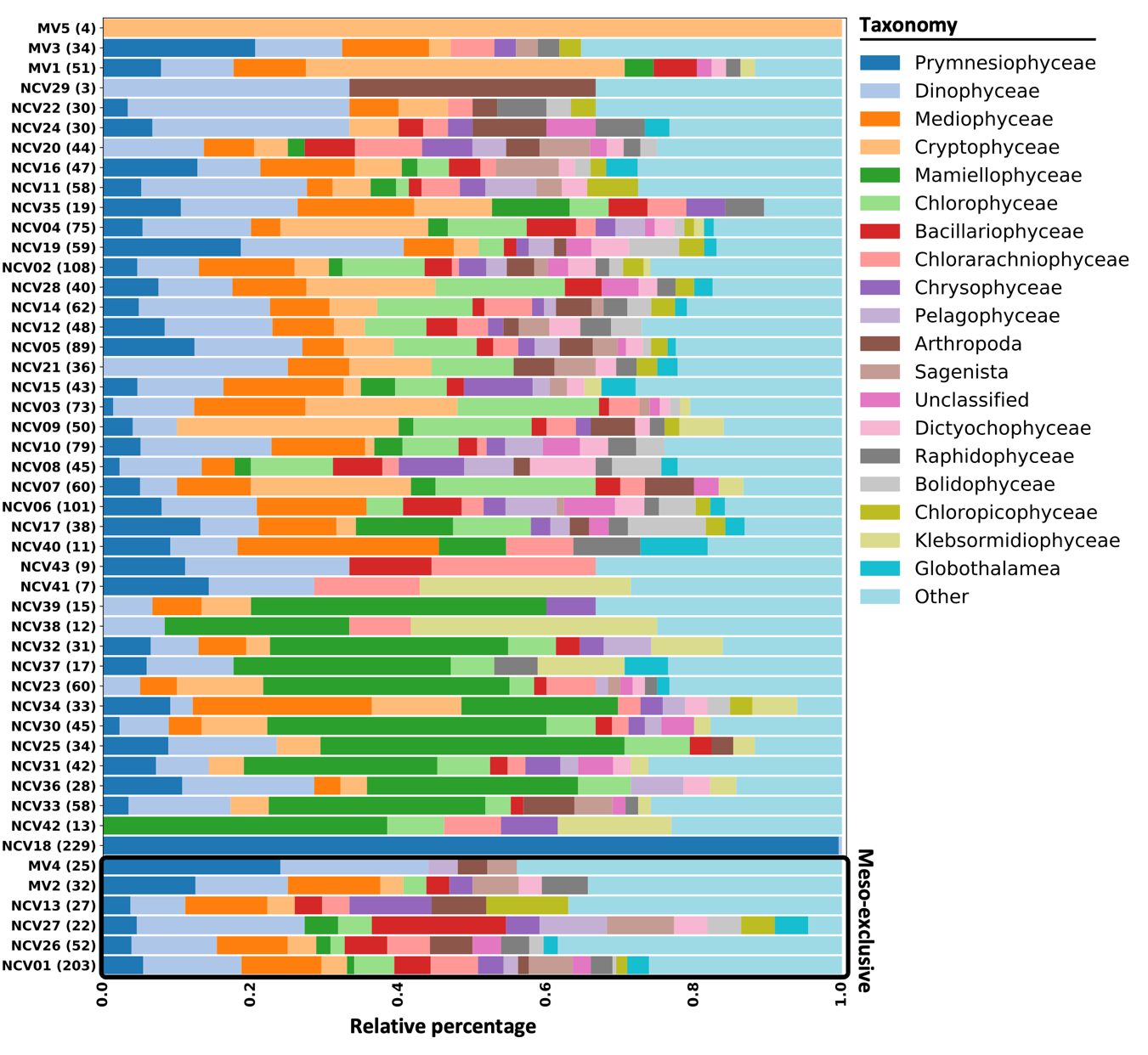
**

**Figure S11. Assignment of eukaryotic-like protein sequences in the Muroto GVMAGs.** Each row represents a Muroto GVMAG. Numbers in parentheses following GVMAG identifiers indicate the total number of genes with hits to eukaryotic taxa in the MarFERRet database. The X-axis shows the relative percentage of gene hits to corresponding eukaryotic classes. GVMAGs enclosed by a black box indicate meso-exclusive GVMAGs.


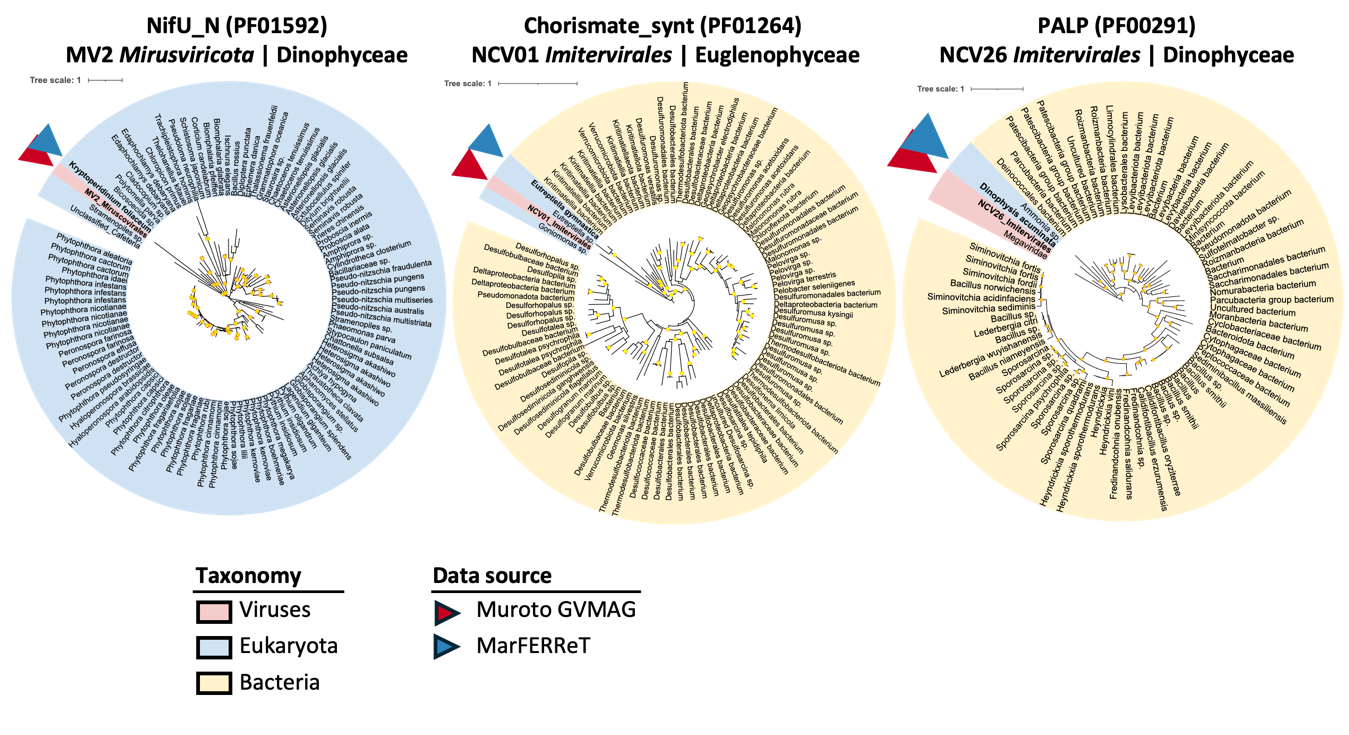


**Figure S12. Phylogenetic trees of eukaryotic-like protein sequences from meso-exclusive GVs and their homologs.** Phylogenetic trees were inferred from protein sequences identified in Muroto GVMAGs and their close homologs retrieved from a combined MarFERReT and NR database. Bootstrap support values >85% are shown. Tip colors denote taxonomic groups (bacteria, eukaryotes, viruses). Blue triangles denote best MarFERReT matches, and red labels highlight proteins from Muroto GVMAG.


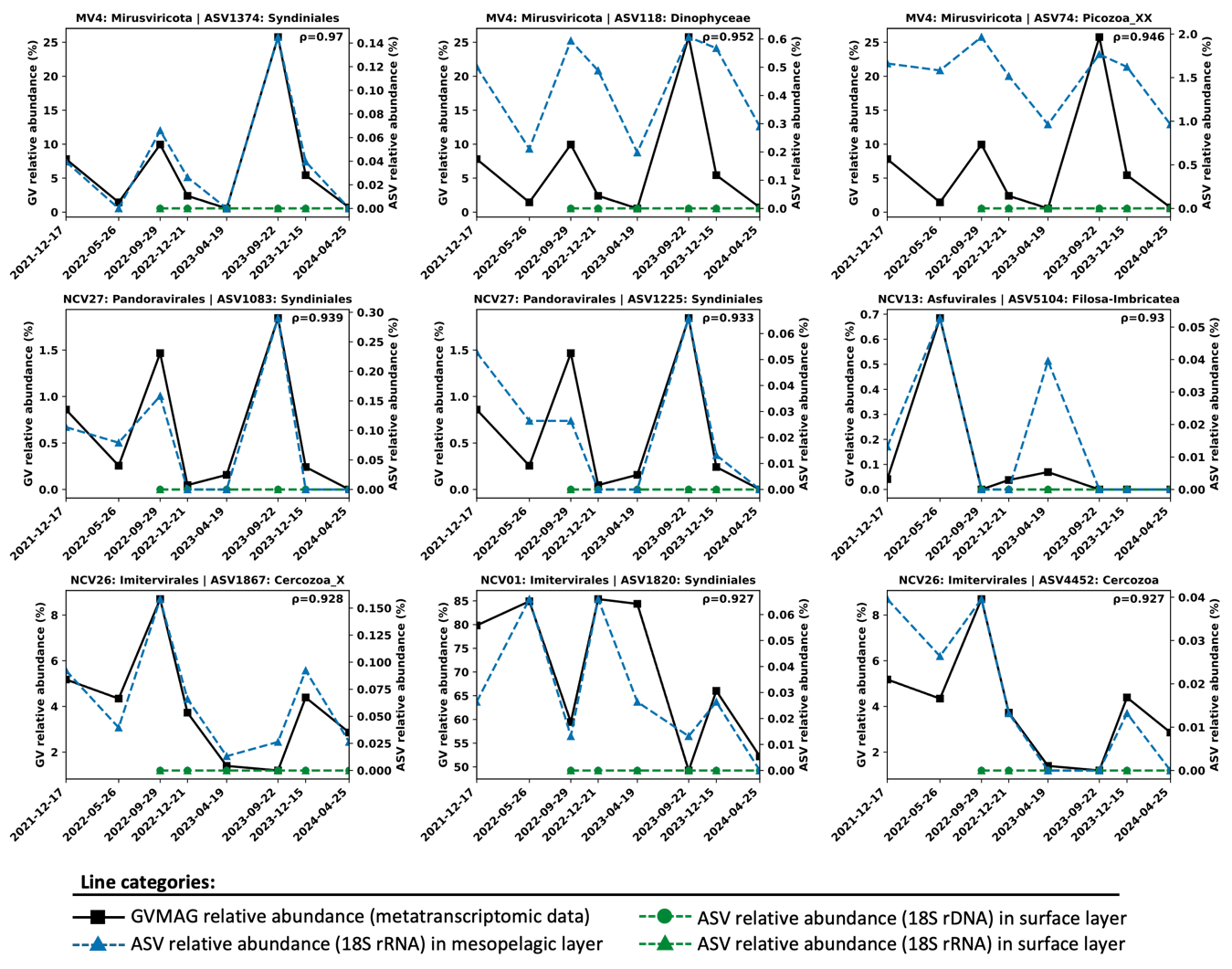


Figure S13. Co-occurring meso-exclusive GVs and microeukaryotes. Spearman’s correlation coefficients were calculated between the relative abundances of meso-exclusive GVs and ASVs in pico- or total-fraction using metatranscriptome and 18S rRNA data from mesopelagic samples (8 paired samples). Significant associations were defined as adjusted *P*-value < 0.05. The relative abundance of ASVs in the surface layers (green line) was added for comparison.

**
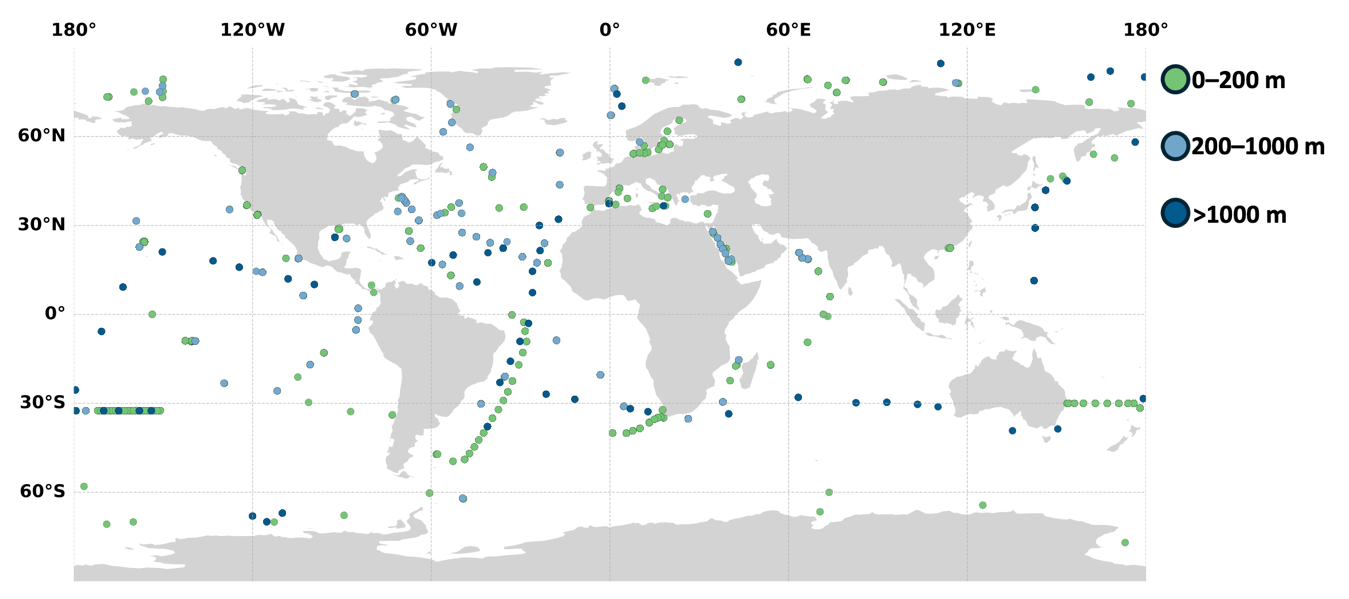
**

**Figure S14. Geographic distribution of 1,890 marine metagenomic datasets used in this study.** Dot colors indicate different depth categories.


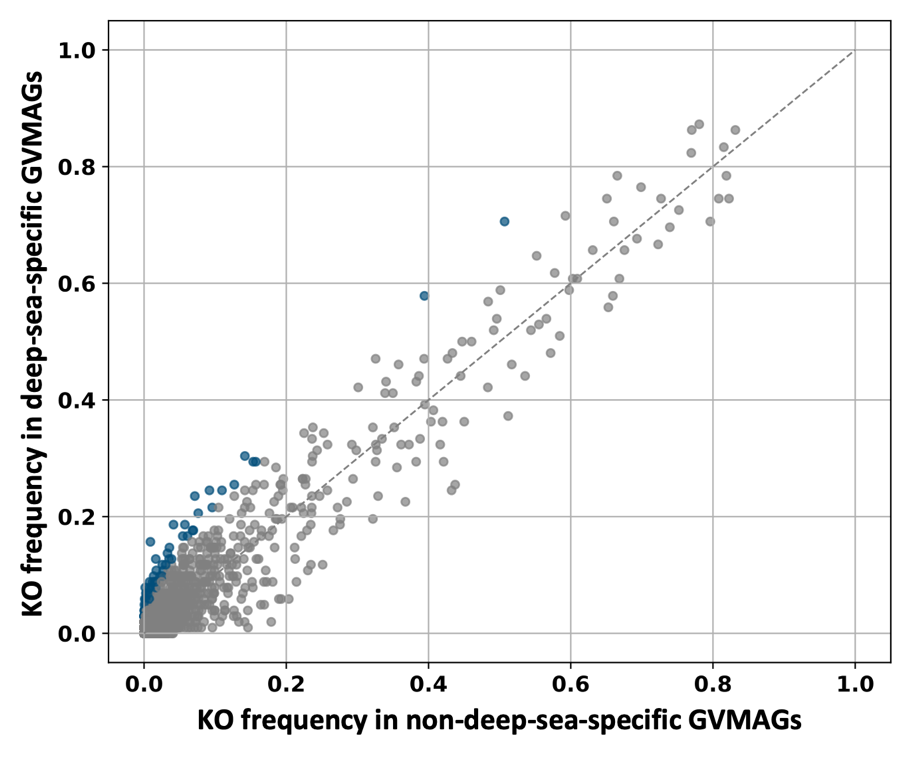


Figure S15. Functional enrichment of deep-sea-specific GVMAGs. Scatterplot comparing mean KO frequencies between deep-sea-specific and other GVMAGs. Each dot represents a KO term, with those enriched in deep-sea-specific GVMAGs highlighted in blue. The dashed diagonal line indicates the 1:1 ratio.


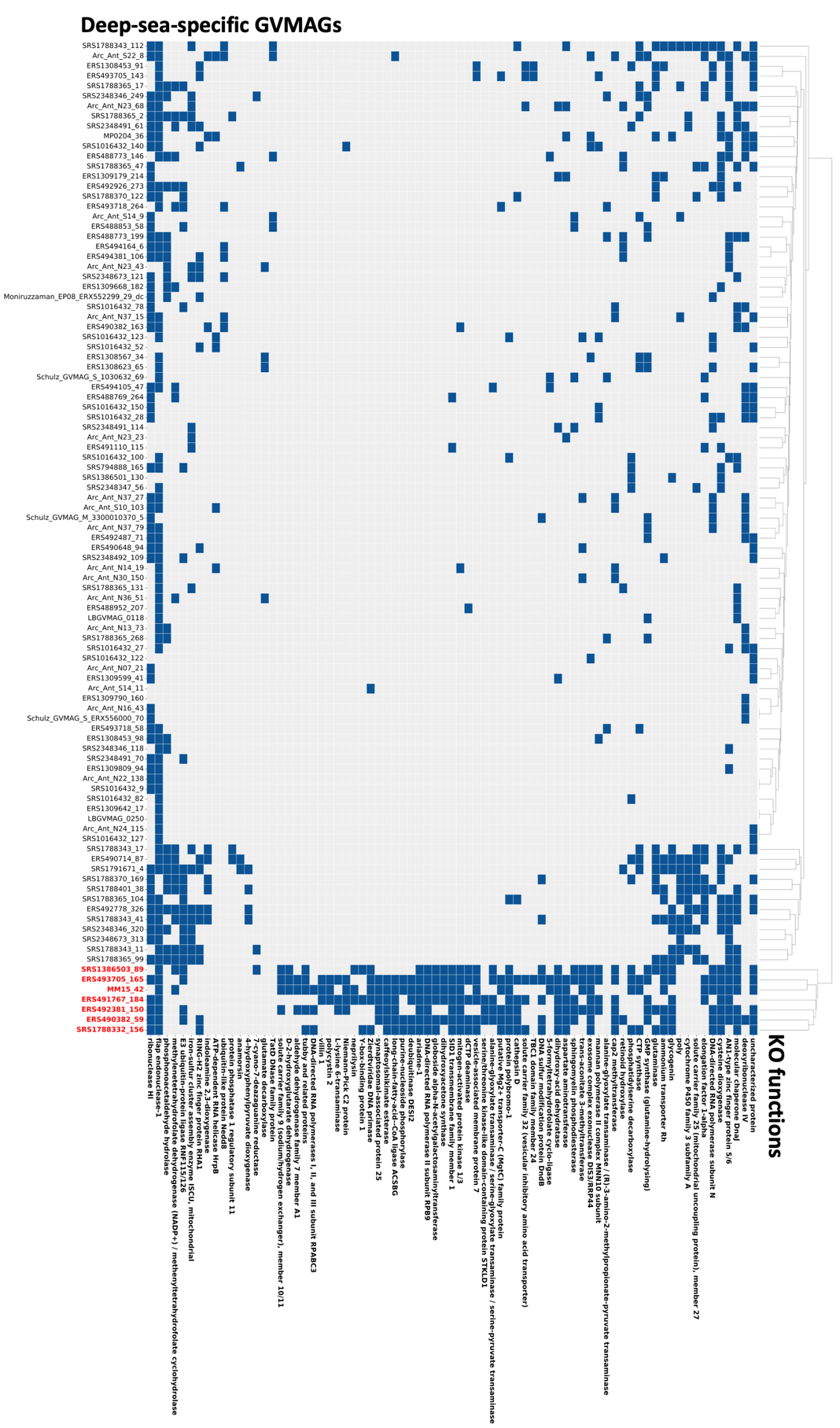


Figure S16. Distribution of deep-sea-specific KOs across deep-sea-specific GVMAGs. Heatmap showing the presence (blue) or absence (gray) of KO terms enriched in deep-sea-specific GVMAGs. Columns represent KO terms, and rows represent deep-sea-specific GVMAGs. MAG names shown in red indicate GVMAGs in the deep-sea-specific GVMAGs clade described in the main text.
